# Bleomycin administered by laser-assisted drug delivery or intradermal needle-injection results in distinct biodistribution patterns in skin: *in vivo* investigations with mass spectrometry imaging

**DOI:** 10.1080/10717544.2021.1933649

**Published:** 2021-06-12

**Authors:** Kristoffer Hendel, Anders C. N. Hansen, Liora Bik, Charlotte Bagger, Martijn B. A. van Doorn, Christian Janfelt, Uffe H. Olesen, Merete Haedersdal, Catharina M. Lerche

**Affiliations:** a Department of Dermatology, Copenhagen University Hospital Bispebjerg and Frederiksberg, Copenhagen, Denmark; b Department of Pharmacy, University of Copenhagen, Copenhagen, Denmark; c Department of Dermatology, Erasmus Medical Center Rotterdam, Rotterdam, The Netherlands

**Keywords:** Skin, bleomycin, ablative fractional laser, laser-assisted drug delivery, drug delivery, topical delivery, intradermal, electroporation, imaging, MALDI, LC-MS, mass spectrometry

## Abstract

Bleomycin (BLM) is being repositioned in dermato-oncology for intralesional and intra-tumoural use. Although conventionally administered by local needle injections (NIs), ablative fractional lasers (AFLs) can facilitate topical BLM delivery. Adding local electroporation (EP) can augment intracellular uptake in the target tissue. Here, we characterize and compare BLM biodistribution patterns, cutaneous pharmacokinetic profiles, and tolerability in an *in vivo* pig model following fractional laser-assisted topical drug delivery and intradermal NI, with and without subsequent EP. *In vivo* pig skin was treated with AFL and topical BLM or NI with BLM, alone or with additional EP, and followed for 1, 2 and 4 h and eventually up to 9 d. BLM biodistribution was assessed by spatiotemporal mass spectrometry imaging. Cutaneous pharmacokinetics were assessed by mass spectrometry quantification and temporal imaging. Tolerability was evaluated by local skin reactions (LSRs) and skin integrity measurements. AFL and NI resulted in distinct BLM biodistributions: AFL resulted in a horizontal belt-shaped BLM distribution along the skin surface, and NI resulted in BLM radiating from the injection site. Cutaneous pharmacokinetic analyses and temporal imaging showed a substantial reduction in BLM concentration within the first few hours following administration. LSRs were tolerable overall, and all interventions permitted almost complete recovery of skin integrity within 9 d. In conclusion, AFL and NI result in distinct cutaneous biodistribution patterns and pharmacokinetic profiles for BLM applied to *in vivo* skin. Evaluation of LSRs showed that both methods were similarly tolerable, and each method has potential for individualized approaches in a clinical setting.

## Introduction

Bleomycin (BLM) is a cytotoxic antitumor agent used for a variety of approved and off-label dermatologic indications including hypertrophic and keloid scars, warts, and non-melanoma skin cancer (Saitta et al., 2008; Bik et al., 2020). Basal cell carcinoma (BCC), the most common type of cancer in the world, is of particular interest as a target for BLM (Campana et al., 2017; Clover et al., 2020). Although conventionally given systemically, BLM is being repositioned in dermato-oncology for intralesional administration (Bik et al., 2020; Clover et al., 2020). Its mechanisms of action include intercalation into DNA and chelation with transition metals to induce reactive oxygen species resulting in DNA damage (Povirk et al., 1979). However, because BLM is highly hydrophilic (log*P*, −7.5) and has a molar mass of 1415 Da, it can neither penetrate the stratum corneum unassisted nor can it easily cross the cell membrane. A drug delivery system is required for topical administration (Hendel et al., 2019).

When used to treat BCCs, intralesional BLM is currently administered by conventional needle injection (NI) to overcome the skin barrier. Several alternative drug delivery systems that may be used to treat BCCs have been tested, including hollow micro-needles (Sabri et al., 2020), pneumatic injection (Erlendsson et al., 2020; Bik et al., 2021; Rosenberg et al., 2021), and laser-assisted drug delivery (LADD) (Wenande et al., 2021). Ablative fractional laser (AFL) has been shown to facilitate and enhance topical BLM delivery via LADD *ex vivo* and is a prime candidate for BLM drug delivery due to its intrinsically therapeutic effect on BCCs (Mirza and Khatri, 2017; Hendel et al., 2019; Navarrete-Dechent, 2019). Laser ablation applied in a low-density fractional pattern creates microscopic laser-channel columns. These columns disrupt the barrier function of the stratum corneum and facilitate drug access (Wenande et al., 2019). In addition, subsequent electroporation (EP) can be applied to augment intracellular uptake (Campana et al., 2017; Gehl et al., 2018; Bik et al., 2020; Clover et al., 2020). Localized application of electric pulses can reversibly permeabilize the cell membrane by inducing transient pores, greatly enhancing uptake and cytotoxicity of BLM (Orlowski et al., 1988; Glass et al., 1997).

Drug biodistribution, cutaneous pharmacokinetics, and tolerability are of paramount importance in the treatment of skin tumors. Cutaneous drug delivery in the skin can be visualized and quantified using mass spectrometry techniques to establish direct spatiotemporal drug uptake patterns without relying on labeling or tagging and confirm that drug biodistribution corresponds to therapeutic targets (Grégoire et al., 2020; Wenande et al., 2021). Matrix-assisted laser desorption/ionization mass spectrometry imaging can visualize relative drug biodistribution in skin sections and liquid chromatography–mass spectrometry can quantify exact concentrations from skin biopsies. Tolerability can be assessed by characterizing local skin reactions (LSRs) and assessing skin integrity by quantifying transepidermal water loss (Olesen et al., 2020). To our knowledge, BLM biodistribution in the skin has not previously been visualized following local administration, and its cutaneous pharmacokinetics have not been established. Our current understanding of the parameters associated with localized BLM administration is limited and is mostly based on clinical efficacy-based observations (Mir et al., 1998; Groselj et al., 2016; Campana et al., 2017).

Here, we characterize and compare BLM biodistribution patterns, cutaneous pharmacokinetic patterns, and local tolerability *in vivo* in porcine skin following LADD and NI, with and without subsequent EP.

## Materials and methods

### Study design

**Figure 1.**
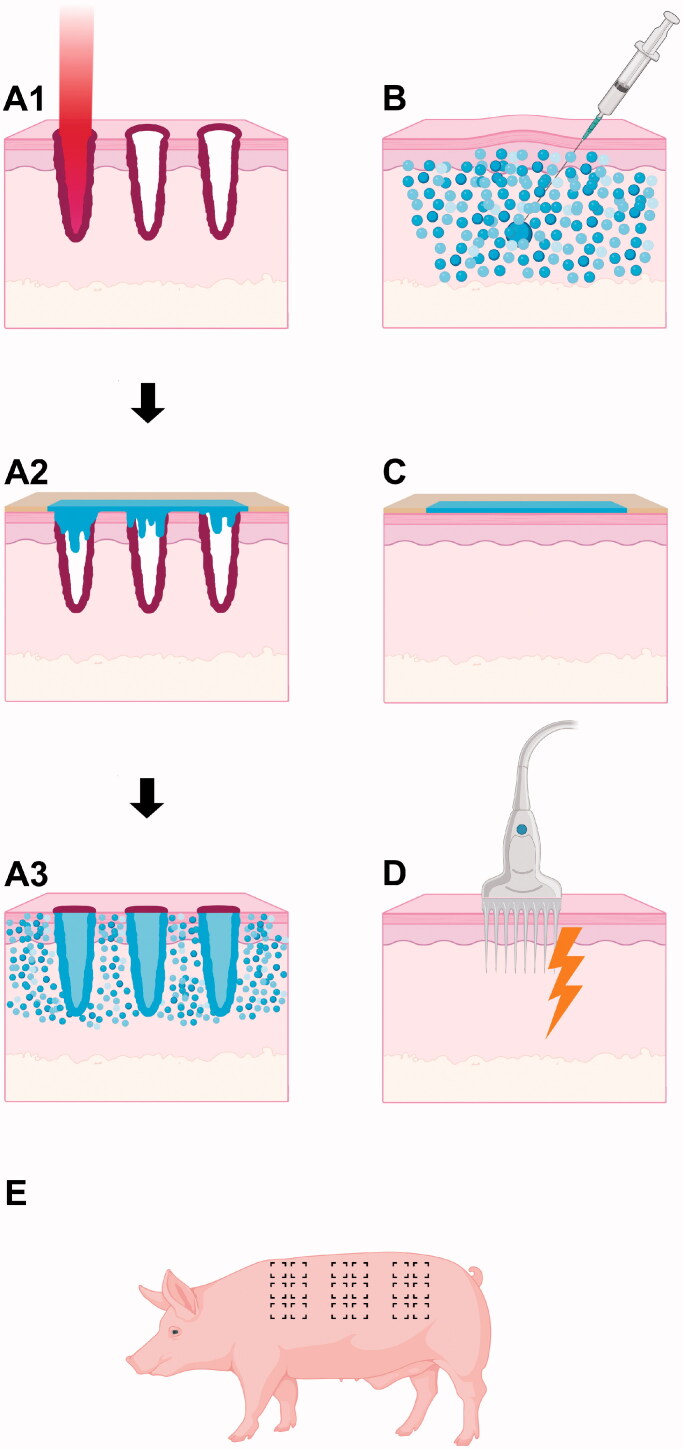
Drug delivery methods. Schematic diagram showing sections of skin and the different drug delivery interventions used in this study. Series A depicts a complete laser-assisted topical drug delivery method consisting of three steps: (A1) Ablative fractional laser (AFL) treatment ablates the skin with precise microbeams to create laser channels. The coagulation zones are shown in dark red; (A2) Topical application on AFL-treated skin. An adhesive well-system is applied to the surface of the lasered skin and filled with a bleomycin (BLM) solution; (A3) The adhesive well-system is removed. BLM builds up in the channels, saturates the coagulation zones, and disperses into the surrounding skin. B: BLM is injected intradermally using a conventional needle and syringe. C: Topical application on intact skin. An adhesive well-system containing BLM is applied to the surface of intact skin. D: Skin is electroporated by inserting a needle probe and firing multiple high-frequency pulses. E: Sample areas were located on the back and flanks of each pig. Interventions were distributed evenly to control for differences in skin thickness. Diagrams are not to scale.

In this *in vivo* pig study, BLM was administered topically with AFL assistance or intradermally using conventional NI, either with or without subsequent EP (Figure 1). Biodistribution, cutaneous pharmacokinetics, and local skin reactions were assessed for up to 9 days (216 h) after exposure. An overview of the interventions and mass spectrometry data is shown in Table 1. The following interventions are shown schematically in Figure 1: AFL (panel A1), AFL + BLM (panels A1–A3), AFL + BLM + EP (panels A1–A3 + D), NI + BLM (panel B), NI + BLM + EP (panels B + D), BLM + EP (panels C + D), and EP (panel D).

**Table 1.** Study design overview.

Drug delivery				Mass spectrometry quantitation and imaging											
*Interventions*				*LC-MS (h) n = 8*							*MALDI (h) n = 1–2*				
Description	Method^a^	BLM	SAL	0	1	2	4	48	144	216	0	1	2	4	48
AFL + BLM	A1–A3	+	–	–	+	+	+	+	+	+	–	+	–	+	+
AFL + BLM + EP	A1–A3 + D	+	–	–	+	+	+	+	+	+	–	+	–	+	+
NI + BLM	B	+	–	+	+	+	+	+	+	+	+	+	–	+	+
NI + BLM + EP	B + D	+	–	–	+	+	+	+	+	+	–	+	–	+	+
BLM + EP	C + D	+	–	–	+	–	–	–	–	–	–	+	–	–	–
AFL	A1	–	–	–	–	–	+	–	–	–	–	–	–	+	–
NI + SAL	B	–	+	–	–	–	+	–	–	–	–	–	–	+	–
EP	D	–	–	–	–	–	+	–	–	–	–	–	–	–	–
				Total LC-MS *n = 232*							Total MALDI *n =* 20				

^a^Method as depicted in Figure 1. AFL: ablative fractional laser; BLM: bleomycin; LC-MS: liquid chromatography–mass spectrometry; MALDI: matrix-assisted laser desorption/ionization; EP: electroporation; NI: needle injection; SAL: saline.

### Animals

The present study was approved by the Danish Animal Inspectorate (license 2017-15-0201-01204) and conducted in accordance with the Federation of European Laboratory Animal Science Associations guidelines. The study included four female gilt pigs (62–75 kg) situated in single confinement with snout access to companion pigs. The pigs acclimatized for two weeks prior to inclusion. General anesthesia was induced using intramuscular benzodiazepine and inhalation of 2% isoflurane and was maintained using isoflurane supported by a bolus of intravenous propofol when required. The pigs were anesthetized up to 4 times (study days 0, 2, 6 and 9). The treatment areas on the back along the spine and down the flanks were carefully shaved to preserve intact skin and 2 × 2-cm sample areas were demarcated with indelible ink, as shown in Figure 1(E). Interventions were performed on similar areas distributed laterally along the spine to accommodate variations in skin thickness down the flanks but were not evenly distributed among the four pigs. At the end of the trial, the pigs were euthanised using intravenous pentobarbital while under general anesthesia.

### Bleomycin preparation

All BLM used was from the same batch (7K062C) and source (Baxter, Deerfield, IL, USA) and was prepared in 0.9% saline (SAL) as a 15,000 IU/mL solution (Hendel et al., 2019).

### Laser treatment

Test fields were exposed to 10,600 nm CO_2_ AFL exposure (Ultrapulse, DeepFx; Lumenis, Inc., Santa Clara, CA, USA) at 250 Hz with 80 mJ/microbeam energies with a 5% density (Figure 1(A1)). These settings reach the mid and deep dermis of pigs of similar size (Olesen et al., 2020), penetrating the skin to a median depth of 1253 µm (interquartile range [IQR]: 966–1437).

### Topical bleomycin application

Topical BLM was applied in customized wells prepared from layered Duoderm hydrocolloid (Convatec, Inc., Greensboro, NC, USA), using Tegaderm (3 M Health Care, St. Paul, MN, USA) as a perforable lid (Figures 1(C) and (A2); Supporting information, Figure S1) (Olesen et al., 2020). The wells were mounted with Klinibond tissue adhesive (Mediq, Utrecht, The Netherlands).

Each well-chamber was loaded with 0.5 mL of BLM solution using a 23-gauge needle without puncturing the skin. Air was retracted from the well-chambers to create a vacuum, effectively creating a homogenous distribution over the test areas. Wells was left on the treatment areas for 1 h before being removed and any visible residual fluid was removed from the skin surface with gauze.

### Needle injections

Injections were performed with 31-gauge needles (BD Veo, Ultra-Fine; Becton, Dickinson and Company, Franklin Lakes, NJ, USA). The skin was penetrated from a 15-degree angle, depositing 0.1 mL BLM intradermally and resulting in an immediate papule, which indicated successful delivery (Figure 1(B)).

### Electroporation

EP was performed with high-frequency pulse (ePore; Mirai Medical, Cork, Ireland) delivering 1300 V/cm at 250 kHz using a probe interface featuring eight needles in a 2 × 4 grid formation (Figure 1(D)). EP was administered 10 min after BLM delivery (NI + BLM + EP) or directly after removal of wells containing BLM (AFL + BLM + EP).

### Tolerability assessment

The skin tolerability of the applied interventions was assessed by scoring LSRs for erythema and edema on a visual and descriptive categorial scale used in similar studies (Wenande et al., 2018; Olesen et al., 2020) and quantifying skin integrity by measuring transepidermal water loss as a function of time (g/m^2^/h) (DermaLab Combo; Cortex Technology, Hadsund, Denmark).

### Sample preparation

Punch biopsies (8 mm) were excised for liquid chromatography-mass spectrometry (LC-MS) quantification and matrix-assisted laser desorption/ionization (MALDI) imaging, snap-frozen on CO_2_ ice and subsequently stored at −80 °C.

Full-skin biopsy samples for LC-MS were dissected into 32 pieces using a scalpel and suspended in 1 mL phosphate-buffered saline. The tissue suspensions were lysed for 30 min (TissueLyser 2; Retsch, Haan, Germany). Next, the homogenates were extracted for 2 h in rotating vials. Samples were centrifuged at 400 RCF for 15 min at 4 °C. Fluid was extracted for LC-MS analyses. Samples for MALDI imaging were vertically cryosectioned at full width (8 mm) in carboxymethylcellulose and fixed on glass slides for analyses.

### LC-MS quantification

The skin homogenates were precipitated by adding 500 µL precipitation solution (2% ZnSO_4_, 25% MeOH) to 250 µL skin homogenate, followed by 10 min centrifugation at 20,000 RCF. Calibration curves were created based on a 0.5 mg/mL BLM stock (Sigma-Aldrich Corp., St. Louis, MO, USA). The stock was diluted to 2000 ng/mL and then diluted 1:1 seven times, to a minimum concentration of 15.625 ng/mL. The eight-point calibration curve was applied at the beginning and end of every sample list. The limit of quantification (LOQ) was 14.49 ng/mL. Samples were analyzed by LC-MS on a Thermo TSQ Vantage triple-quadrupole mass spectrometer equipped with a Thermo Accela high-performance liquid chromatography system (Thermo Fisher Scientific, Waltham, MA, USA). A 100 × 2.1 mm Kinetex 2.6 µ XB-C18 100 A column was used with isocratic elution (solvent composition: 8% MeOH, 0.1% Formic acid, and 91.9% H_2_O). The transition pairs used for quantitation were *m/z* 713.5 to 530.0 for BLM B2 and *m/z* 708.0 to 493.5 for BLM A2.

### MALDI imaging

The skin samples were dried in a vacuum desiccator for 30 min prior to coating with the matrix. The matrix (30 mg/mL 2,5-dihydroxybenzoic acid; 90% MeOH; 10% H_2_O) was applied using an iMatrix sprayer and the following settings: height, 80 mm; width, 40 mm; depth, 40 mm; line distance, 1 mm; speed, 90 mm/s; density, 3 µL/cm^3^; and 12 cycles. Mass spectrometry imaging was performed on SMALDI5 and SMALDI10 (TransMIT, Giessen, Germany) Q-Exactive systems, using either line or pixel mode. The images were acquired at a mass resolution of 140,000 at 200 *m/z* and a scan range of 225–1750 *m/z*. The acquired Thermo RAW files were converted to imzML format using RawToIMZML, and the images were generated in MSiReader (ver. 1.02; North Carolina State University, Raleigh, NC, USA). The phospholipid PC signal (36:4) detected as its H^+^ adduct at *m/z* 782.56939 was used as a tissue biomarker for the skin, and BLM B2 was detected at *m/z* 1425.51881.

### Units and calculations

Skin BLM concentrations presented in µg/cm^3^ were based on the absolute quantity of BLM present in the sample relative to sample weight and a density of 1.09 g/cm^3^ (White et al., 1992). Concentrations given in IU/cm^3^ were calculated based on the applied BLM formula with a concentration of 15,000 IU/mL equivalent to 7.06 mg/mL (Hendel et al., 2019).

### Statistics

Descriptive statistics are presented as medians and IQR. Non-parametric Mann–Whitney tests were used throughout a 5% alpha level and corrected with Holm–Bonferroni *posthoc* tests when applicable. The sample size was estimated based on *in vitro* studies with a standardized difference of 1.5 and a 20% beta. Analyses were computed using SPSS (ver. 27; International Business Machines Corp, Armonk, NY, USA) and plotted using Prism 8 software (ver. 8; GraphPad, San Diego, CA, USA).

## Results

### Biodistribution by MALDI imaging

**Figure 2.**
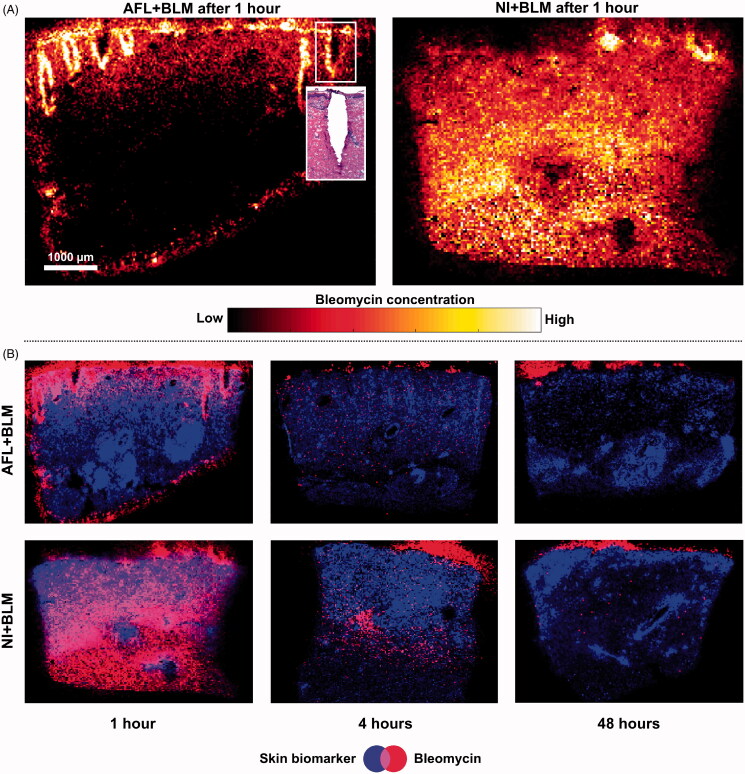
Treated skin samples for BLM B2 (*m/z* 1425.56323) and a skin-tissue biomarker (phospholipid) visualized using matrix-assisted laser desorption/ionization mass spectrometry imaging. A: BLM biodistribution following laser-assisted drug delivery (LADD; left panel) and needle injection (NI; right panel). The most intense white regions indicate the strongest BLM signal. Each image shows contrasts in signal intensity within one image and BLM quantities cannot be compared between the two images. The left panel shows a section of a laser channel that has been stained with hematoxylin and eosin. B: A series of images for LADD (top panels) and NI (bottom panels) at 1, 4 and 48 h depicting BLM and a skin-tissue biomarker. AFL: ablative fractional laser; BLM: bleomycin; EP: electroporation; NI: needle injection; LADD: laser-assisted drug delivery.

Imaging revealed that both laser-assisted (AFL + BLM) and needle-injection (NI + BLM) delivery methods resulted in detectable intradermal concentrations of BLM. The spatial resolution showed that AFL + BLM led to BLM accumulating in the coagulation zones, dispersing into the surrounding tissue, and forming a horizontal belt-like shape that was limited in depth by the laser-channel dimensions (Figure 2(A), left panel). In contrast, NI + BLM led to homogenous and widespread BLM distribution after 1 h (Figure 2(A), right panel), although temporal resolution showed that BLM had radiated outwards from a primary injection point after 4 h (Figure 2(B), 1–4 h). All images showed residual BLM present on the skin surface.

Images of EP and non-EP samples were similar. For example, there were no differences between AFL + BLM + EP and AFL + BLM images. The BLM + EP control intervention resulted in negligible traces of BLM on the skin surface. All BLM-negative control images were negative for BLM (Supporting information, Figure S2).

### Quantified cutaneous pharmacokinetics by LC-MS

**Figure 3.**
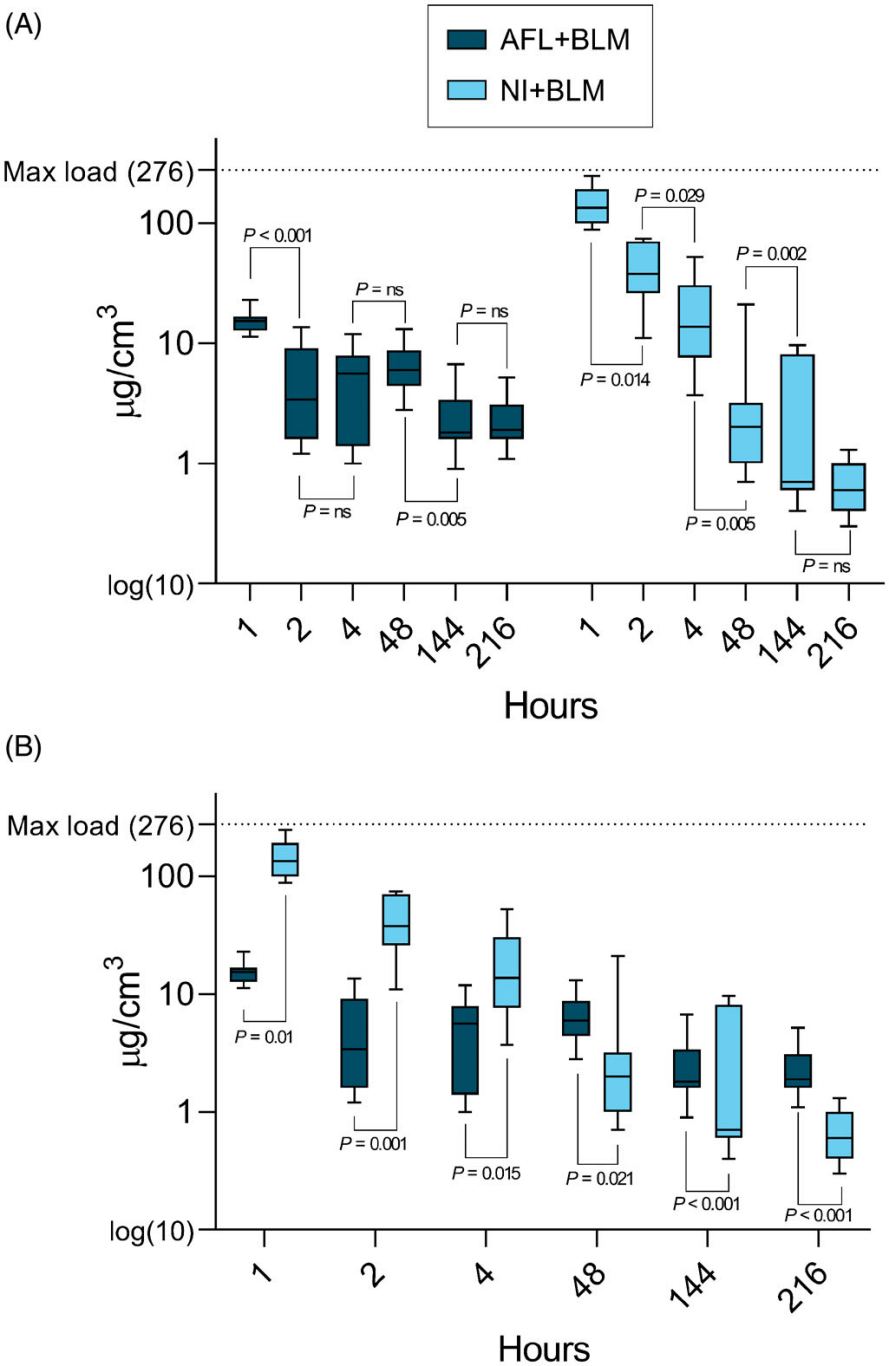
Box and whisker (min–max) plots of BLM concentrations. BLM concentrations quantified by liquid chromatography-mass spectrometry and visualized using log(10) box plots with quartile boxes and min–max whiskers. The maximum-load line marks the experimentally determined maximum BLM saturation per cubic centimeter of skin. Quantified BLM is not necessarily situated intradermally—please review Table 2 for spatial distribution data. A: LADD and NI grouped by time. B: Interleaved plot of the same data comparing concentrations between the two interventions. Abbreviations: AFL: ablative fractional laser; BLM: aleomycin; EP: electroporation; NI: needle injection; LADD: laser-assisted drug delivery.

Punch biopsies taken immediately after BLM injection (NI + BLM, 0 h) had 276.1 µg/cm^3^ (IQR: 210.9–357.7) of BLM, which was the maximum BLM load observed in this study and saturated a full-skin biopsy. In comparison, after 1 h, AFL + BLM resulted in 15.4 µg/cm^3^ and NI + BLM in 133.6 µg/cm^3^ of BLM, equivalent to 5.5% and 48.3% of the maximum load, respectively (Table 2). Low concentrations of BLM were detected following AFL + BLM after 4 h, but spatiotemporal imaging suggested that this BLM was present on the skin surface. For NI + BLM, the intradermal BLM concentration decreased significantly over the period of 1 to 4 h (133.6 to 2.0 µg/cm^3^; *p <* .05), as shown in Figure 3. Adding EP did not significantly change the intradermal BLM concentrations for any intervention at any time point. In particular, neither the BLM concentrations in AFL + BLM and AFL + BLM + EP samples nor those in NI + BLM and NI + BLM + EP samples were significantly different. As expected, LC-MS results for all BLM-negative controls (AFL, NI + SAL, and EP alone) were negative (Table 2).

**Table 2.** LC-MS quantification of bleomycin drug delivery in skin.

		Quantified (LC-MS)		Imaged (MSI)	Calculated	
Adm.	Time	µg/cm^3^	IQR	Spatial placement	IU/cm^3^	% of max^a^
AFL + BLM	1 h	15.4	(12.7–16.8)	Intradermal	32.6	5.5
	2 h	3.4	(1.6–9.1)	—	7.2	1.2
	4 h	5.6	(1.4–7.9)	Surface only	11.9	2.0
	48 h	6.0	(4.4–8.7)	Surface only	12.7	2.1
	144 h	1.8	(1.6–3.4)	—	3.8	0.6
	216 h	1.9	(1.6–3.1)	—	4.0	0.6
						
NI + BLM	0 h	276.1	(210.9–357.7)	Intradermal	584.5	100.0
	1 h	133.6	(99.5–192.0)	Intradermal	283.2	48.3
	2 h	37.8	(26.1–71.0)	—	80.1	13.6
	4 h	13.7	(7.7–30.3)	Intradermal	29.0	4.9
	48 h	2.0	(1.0–3.2)	Surface only	4.2	0.7
	144 h	0.7	(0.6–0.8)	—	1.5	0.2
	216 h	0.6	(0.4–1.0)	—	1.3	0.2
						
BLM + EP	1 h	4.7	(4.2–7.8)	Surface only	9.9	1.7
AFL	4 h	0	(0–0)	Not detected	0	0
NI + SAL	4 h	0	(0–0)	Not detected	0	0
EP	4 h	0	(0–0)	Not detected	0	0

^a^Maximum concentration is calculated from the saturation limit of BLM in the skin (NI + BLM at 0 h). AFL: ablative fractional laser; BLM: bleomycin; LC-MS: liquid chromatography–mass spectrometry ;MSI: mass spectrometry imaging; IQR: interquartile range (Q1–Q3); IU: international units; EP: electroporation; NI: needle injection; SAL: saline.

—: Not included for testing.

### Tolerability and safety

**Figure 4.**
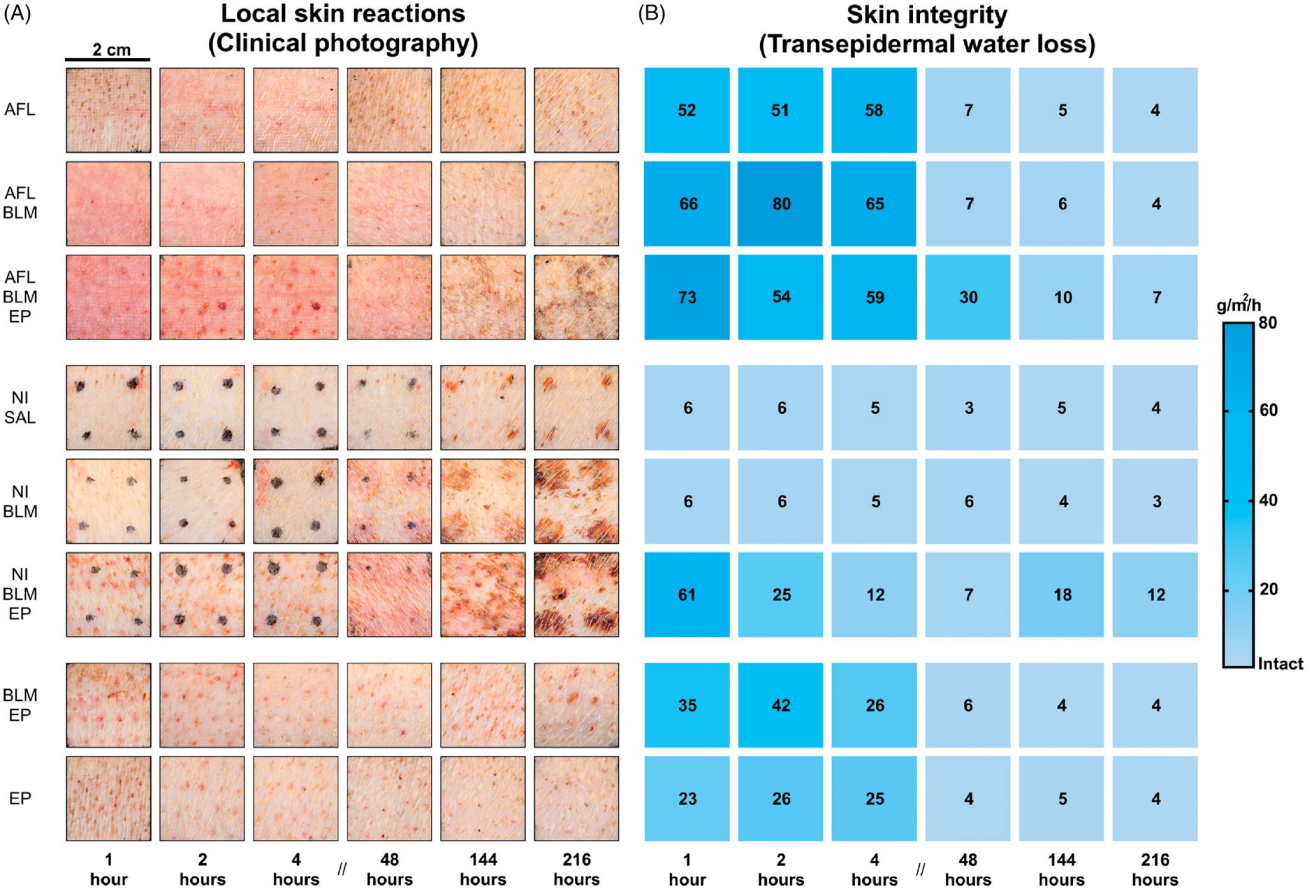
Local skin reactions and skin integrity measurements. A: Local skin reactions are shown using clinical photography. Laser-channel grids can be seen in the first block, covering the laser-based interventions. The second block shows black dots marking the papule endpoint spots for the NI interventions. The third block shows topical BLM and electroporation controls for the laser and NI interventions. A grid of needle entry points can be seen on all EP-based interventions. B: Skin integrity heatmap based on transepidermal water loss (g/m^2^/h). AFL: ablative fractional laser; BLM: bleomycin; EP: electroporation; NI: needle injection; SAL: saline.

Overall, the interventions were well tolerated with only mild to moderate LSRs (Figure 4(A)). AFL-based interventions induced the most intense erythema in the first few hours after treatment (*p* = .003) but the laser-exposed skin gradually recovered over 9 d, as shown by the clinical photographs in Figure 4(A). In contrast, NI did not generate distinct LSRs, except for mild edema within the first few hours, and no differences were observed between NI + SAL and NI + BLM interventions. However, 2 d after NI + BLM and NI + BLM + EP treatments, LSRs became more intense and the treatments that included BLM showed significantly more intense LSRs than did the SAL controls (*p* = .029). EP alone resulted in mild erythema, but also aggravated erythema when combined as AFL + BLM + EP or NI + BLM + EP treatments. Moderately intense LSRs following NI + BLM + EP appeared concurrently with a substantial loss of skin integrity, and this was the only intervention in which skin integrity did not completely recover within 9 d (*p* < .001; Figure 4(B)). LC-MS analyses found no systemic uptake of BLM in blood samples from two pigs treated only with AFL-based interventions. Blood samples from two pigs subject to both AFL- and NI-based interventions were positive for BLM in the hours after initial administration. LC-MS revealed systemic uptake of BLM in these pigs’ 1-, 2- and 4-h samples at 1.21, 0.73 and 0.63 µg/mL, respectively, whereas 2-, 6- and 9-d samples were negative for BLM.

## Discussion

The main challenge for topical administration of BLM is the large molecular size preventing skin penetration (Morrow et al., 2007; Hendel et al., 2019), whereas cutaneous biodistribution and pharmacokinetics are the main concerns in the shift from systemic to localized administration (Mir et al., 1998; 2006; Gehl et al., 2018). In this study, mass spectrometry imaging allowed direct spatiotemporal characterization of BLM biodistribution and pharmacokinetics, and mass spectrometry determined BLM concentrations in full-skin biopsies (Grégoire et al., 2020). We found that both AFL + BLM and NI + BLM successfully delivered BLM intradermally, resulted in effective BLM biodistribution, with good local skin tolerability. Interestingly, spatiotemporal imaging had striking consequences for the interpretation of BLM pharmacokinetics in skin. Mass spectrometry imaging put the quantitative evidence into a qualitative perspective, revealing that the BLM quantified by LC-MS was not necessarily situated intradermally.

As expected, the concentration of BLM present after 1 h depending on the administration route, with NI + BLM delivering 5-fold higher concentrations of BLM than AFL + BLM. This difference reflects the nature of the delivery methods when delivered concentrations of BLM (µg/cm^3^) are expressed as a proportion of applied concentrations (µg/mL). NI + BLM results in most of the BLM applied being deposited in the skin, whereas AFL + BLM only deposit a fraction of the applied volume, rendering direct comparisons very difficult. Regardless, both delivery methods deposited high concentrations of BLM. A recent study that used mass spectrometry to quantify BLM in human tumor tissue following intravenous administration found that approximately 0.1 µg/g of BLM was present 8 min after infusion (Kosjek et al., 2016). In comparison, a substantially higher concentration of 15.4 µg/cm^3^ BLM was present 1 h after administration of AFL + BLM in healthy pig skin.

In our study, BLM was present in samples for the entire study duration of 9 d, regardless of the administration route. However, intradermal BLM was washed out within 4 h after AFL + BLM, whereas an insignificant quantity of BLM remained 4 h after NI + BLM. Intradermal drug residence time is essential when supplementing treatments with EP: the current consensus on BCC treatment is that EP should follow within minutes of BLM administration (Mir et al., 2006; Gehl et al., 2018; Rotunno et al., 2018), but our results indicate that high concentrations of BLM are present in the tissue for at least 1 h. Expectedly, at the available spatiotemporal resolution, interventions that included EP were indistinguishable from interventions that did not i.e. images of AFL + BLM and AFL + BLM + EP treatments were similar. Enhanced intracellular uptake following EP could have affected the BLM tissue residence times, but this was not apparent from the temporal imaging or quantitative results (*p* > .05).

All treatments were well tolerated with moderate acute LSRs and almost complete recovery of skin integrity within 216 h. AFL + BLM + EP caused the most intense acute LSRs and loss of skin integrity, but NI + BLM + EP exhibited a later onset of LSRs and a longer-lasting impact. These observations reflect the main differences between the two approaches. AFL causes acute tissue damage with an immediate response, possibly concealing otherwise visible LSRs of BLM. NI + BLM causes BLM-mediated effects, whereas the NI itself results in negligible skin damage. The increased intensity of the late-onset LSRs visible after NI + BLM + EP is probably attributable to an increased inflammatory response exacerbated by higher intracellular uptake of BLM—it is unlikely that the needle trauma itself would have provoked such a response. Overall, local skin tolerability was not a major concern. Blood samples from two out of four pigs had detectable BLM concentrations at 1, 2 and 4 h. The highest concentration of BLM measured in the blood samples was 1.48 µg/mL at 1 h after the first intervention. In comparison, a pharmacokinetic study in humans reported a concentration of 1.42 µg/mL BLM 40 min after intravenous injection of up to 30,000 IU of BLM (Groselj et al., 2016). The BLM in blood samples from our study was probably derived from percutaneous systemic uptake from the high load of more than 225,000 IU BLM delivered by NI alone. As such, it is unlikely to be a meaningful limitation for clinical use. Interestingly, a high BLM load was also administered to the two pigs treated with AFL + BLM but not NIs; however, no BLM was detected in blood samples from these pigs at any time point.

The AFL treatment in this study was tailored to reach the mid-deep dermis (reaching at least 1000 µm). We applied 80 mJ/mb and reached a median depth of 1253 µm. This fits well with a previous study using in vivo pig skin where 80 mJ/mb created laser channels of ∼1300 µm depth (Olesen et al., 2020). Reaching a similar depth in human skin may require lower settings, as 40 mJ/mb has been shown to reach a depth of ∼900 µm in human skin during a trial on drug delivery (Wenande et al., 2021). In addition, in a clinical setting local anesthesia can mitigate the discomfort of fractional ablation applied to reach the target depths (Wenande et al., 2021).

**Figure 5.**
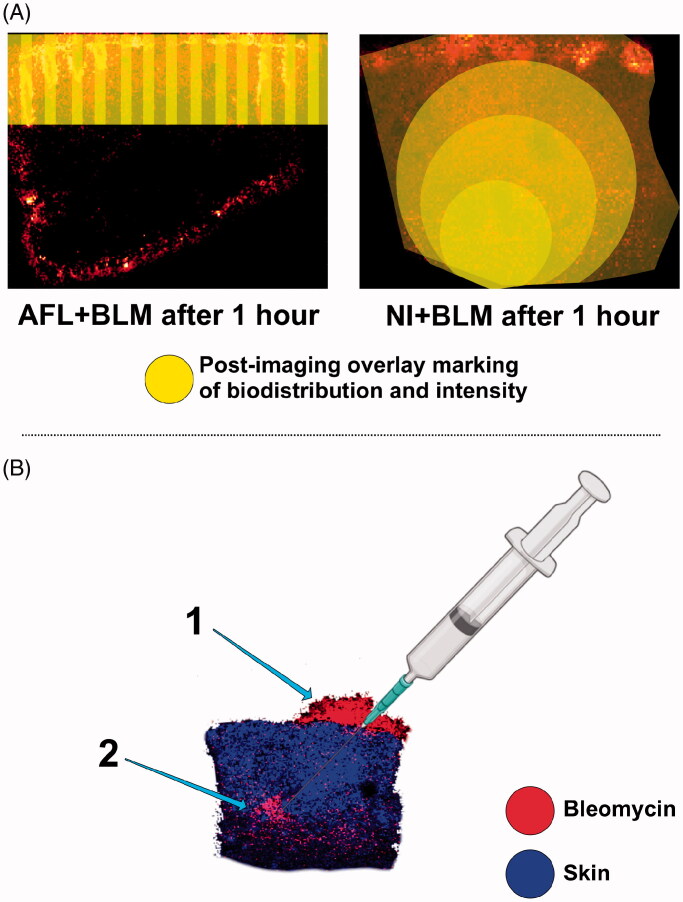
Biodistributionresults interpreted as an overlay on MALDI imaging. A: Biodistribution patterns visualized as post-imaging overlay markings on matrix-assisted laser desorption/ionization images (the original data are shown in Figure 2). AFL + BLM results in a belt-shaped delivery zone, with laser-channel coagulation zones showing the highest concentrations of BLM. NI-BLM shows that the peak BLM concentration is centered on the primary injection point with BLM radiating outwards. B: Example showing that the strongest BLM signal 4 h after NI corresponds to the initial injection site, with almost no residual BLM in the surrounding tissue; 1: BLM remaining on the surface of the skin; 2: Initial point of deposition. The skin-tissue biomarker is shown in blue and BLM is shown in red. MALDI: Matrix-assisted laser desorption/ionization; AFL: Ablative fractional laser; BLM: Bleomycin; NI: Needle injection.

Distinct biodistribution patterns were visualized by mass spectrometry imaging (Figure 2) and are reconceptualised in Figure 5(A), showing individualized approaches to treating skin tumors. AFL + BLM could be a promising method for treating superficial BCCs and the depth of channel penetration may be adjusted by changing the laser energy settings. In addition, AFL has intrinsic therapeutic effects. NI + BLM can deliver high concentrations of BLM which disperse outwards from the initial point of delivery, and this approach may be well-suited for treating deep nodular lesions. Furthermore, although cutaneous tumors are prime targets for BLM, other indications may benefit from AFL + BLM and AFL + BLM + EP. A recent review, which describes alternative delivery routes such as microneedle pens and patches, highlights recalcitrant warts as targets for BLM amongst other indications such as keloid and hypertrophic scars (Bik et al., 2020). Many BLM targets may benefit from AFL treatment alone (Cavalié et al., 2015; Tan et al., 2021) and may respond favorably to the combination of AFL + BLM (Bik et al., 2020). Clinical applications of this technology may also be enhanced if different energy settings could be used for AFL treatments. Adjusting AFL energy-levels can control the penetration depth and the resulting laser-channel dimensions (Wenande et al., 2019). An *ex vivo* study has shown that laser-channel dimensions alter BLM penetration depth (Hendel et al., 2019), but this has not been demonstrated *in vivo*.

In conclusion, AFL and NI result in distinct but effective cutaneous biodistribution patterns and pharmacokinetic profiles for BLM applied to *in vivo* skin. Evaluation of LSRs showed that both methods were well tolerated, and each method has potential for different indications and individualized approaches in a clinical setting.

## Supplementary Material

Supplemental Material
