# Supplementary material for: Bleomycin administered by laser-assisted drug delivery or intradermal needle-injection results in distinct biodistribution patterns in skin: in vivo investigations with mass spectrometry imaging
Source: Drug Deliv. 2021 Jun 12;28(1):1141–9. doi: 10.1080/10717544.2021.1933649 (PMC8205002; doi:10.1080/10717544.2021.1933649)
Supplement: Supplemental Material [file IDRD_A_1933649_SM7930.zip › Supporting_information.docx]

**Supporting information**

**Figure S1:** Schematics for customised wells for topical drug application. Duoderm hydrocolloid (15 x 15 x 0.1 cm) was used for well material. Tegaderm transparent medical dressing film was used as a lid for the wells. A: Three Duoderm hydrocolloid were stacked to increase well depth to 0.3 cm in total. B: 2 x 2 cm wells were cut by scalpel with a 1.5 cm border between wells. Subsequently, a film dressing is applied on top to lid the wells (not depicted).

**Figure S2**: Skin samples imaged for BLM B2 (*m/z* 1425.56323) and a skin-tissue biomarker (phospholipid) visualised using matrix assisted laser desorption/ionisation mass spectrometry imaging. Red colour is bleomycin. Blue colour is the biomarker. Images are within-relative; colour intensity and bleomycin quantities cannot be compared between images. AFL: Ablative fractional laser; BLM: Bleomycin; EP: Electroporation; NI: Needle injection; SAL: saline.
