# Supplementary figures and images for: Bleomycin administered by laser-assisted drug delivery or intradermal needle-injection results in distinct biodistribution patterns in skin: in vivo investigations with mass spectrometry imaging
Source: Drug Deliv. 2021 Jun 12;28(1):1141–9. doi: 10.1080/10717544.2021.1933649 (PMC8205002; doi:10.1080/10717544.2021.1933649)

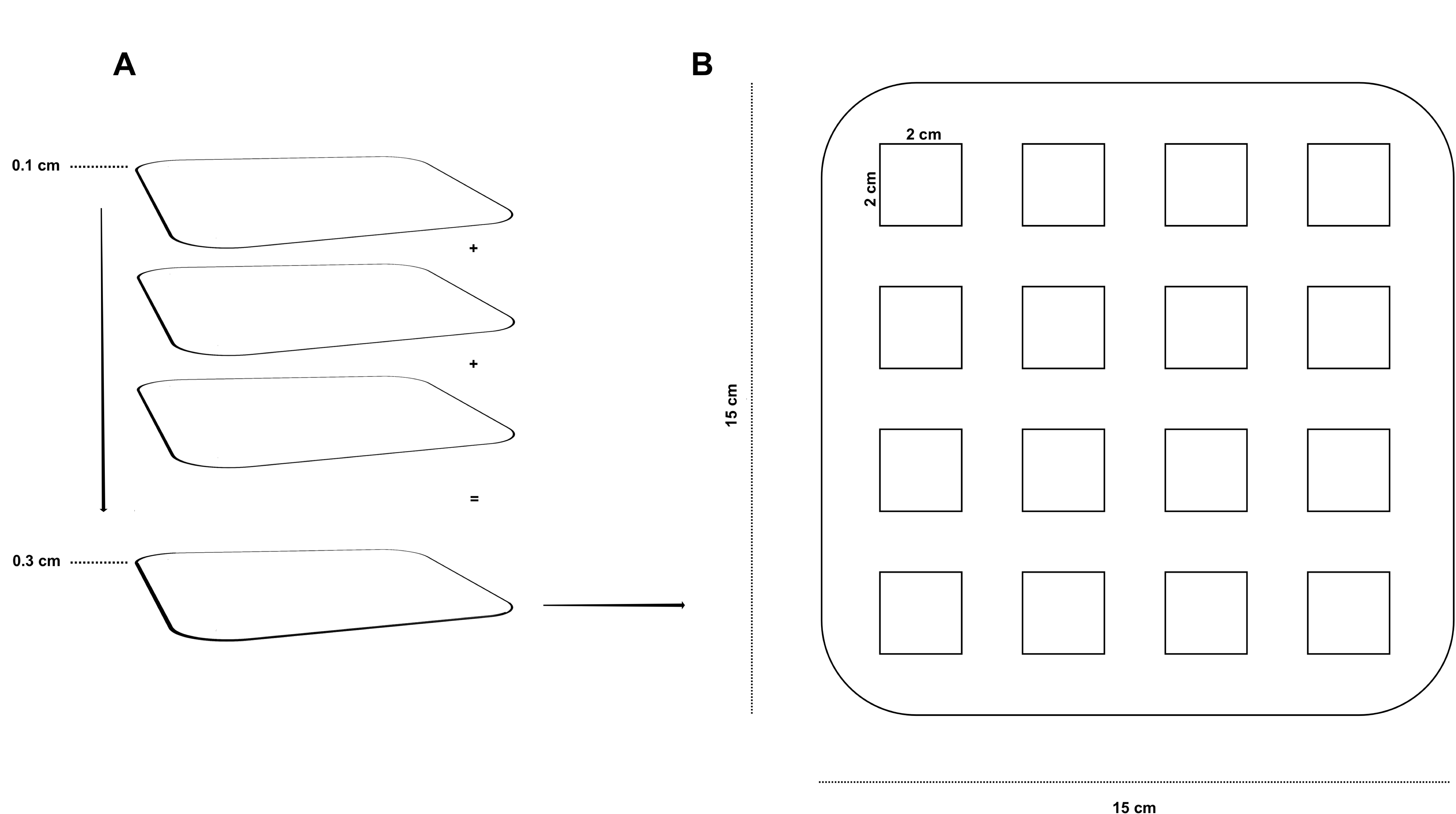

Supplement: Supplemental Material [file IDRD_A_1933649_SM7930.zip › Supporting_FigureS1.png]

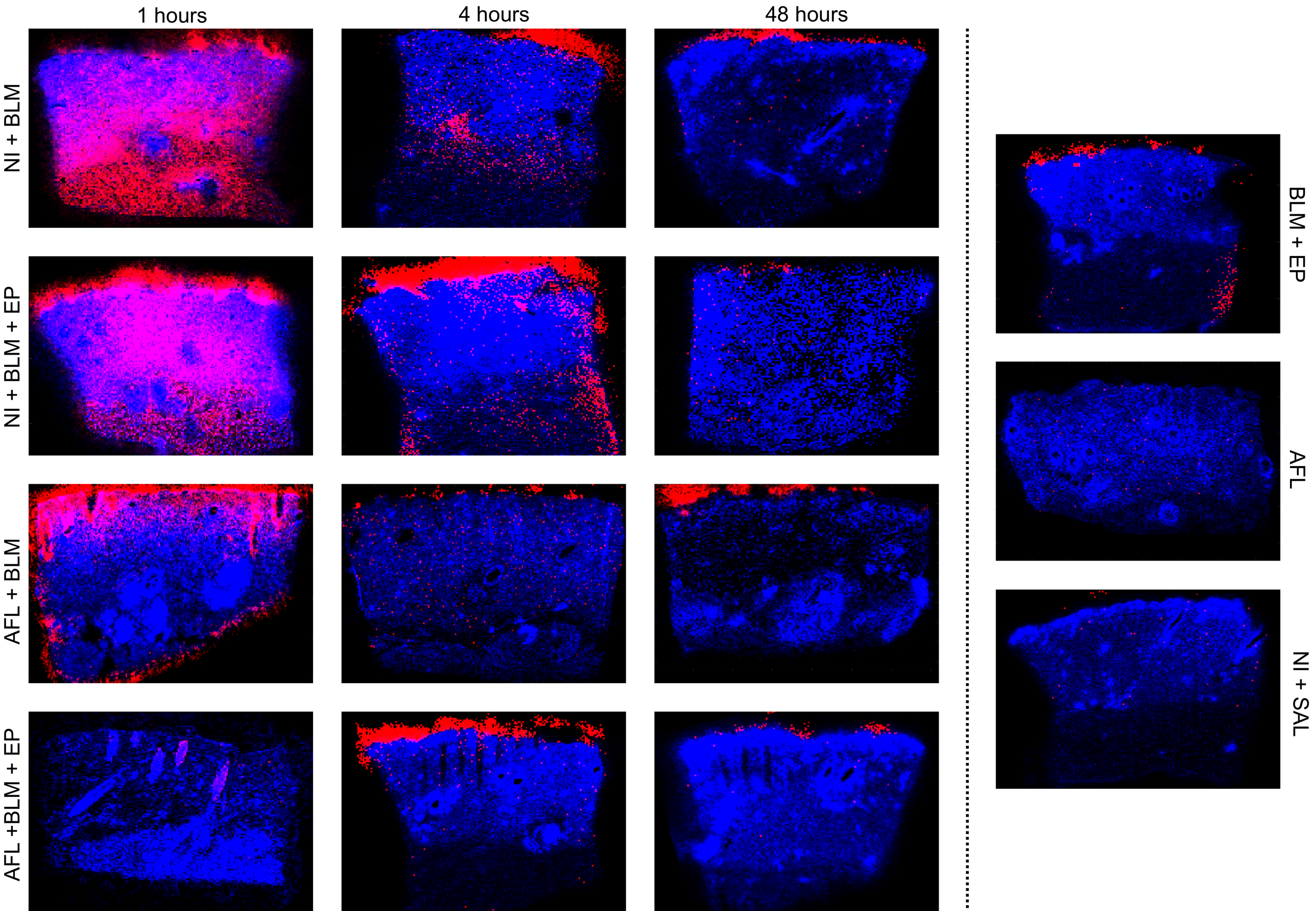

Supplement: Supplemental Material [file IDRD_A_1933649_SM7930.zip › Supporting_FigureS2.png]
